# The Status of Vitamin B12 and Folate among Chinese Women: A Population-Based Cross-Sectional Study in Northwest China

**DOI:** 10.1371/journal.pone.0112586

**Published:** 2014-11-12

**Authors:** Shaonong Dang, Hong Yan, Lingxia Zeng, Quanli Wang, Qiang Li, Shengbin Xiao, Xiaojing Fan

**Affiliations:** Department of Epidemiology and Health Statistics, School of Public Health, Xi'an Jiaotong University Health Science Center, Xi'an, Shaanxi, People's Republic of China; CSIR-INSTITUTE OF GENOMICS AND INTEGRATIVE BIOLOGY, India

## Abstract

**Objective:**

To assess the status of the vitamin B12 and folate of Chinese women living in northwest China.

**Methods:**

A population-based cross-sectional study was conducted in 2008 among Chinese women aged 10–49 years living in Shaanxi province of northwest China. A stratified multistage random sampling method was adopted to obtain a sample of 1170 women. The women were interviewed for collection of their background information and their plasma vitamin B12 and folate were measured with the immunoassay method. The status of both vitamins was evaluated and the prevalence of deficiency was estimated.

**Results:**

The median value of the women was 214.5 pg/mL for vitamin B12 and 4.6 ng/mL for folate. The urban women had a significantly higher vitamin B12 (254.1 vs. 195.9 pg/mL) but lower folate (4.4 vs. 4.7 ng/mL) than rural women. Total prevalence of deficiency was 45.5% (95% CI: 42.6%∼48.4%) for vitamin B12 and 14.7% (95% CI: 12.6%∼16.8%) for folate. About 36% of women presented vitamin B12 deficiency alone, 5.2% belonged to folate deficiency alone and 9.5% was combined deficiency in both vitamins. More than 25% of the women were in marginal vitamin B12 status (200–299 pg/mL) and 60% in marginal status of folate (3–6 ng/mL). About 75.2% of rural women with folate deficiency were deficient in vitamin B12 and 46% for urban women. Quantile regression model found decreasing coefficient of folate status across 73 different quantiles of vitamin B12, which indicated that the women with folate deficiency had lower vitamin B12 significantly compared with those with no deficiency.

**Conclusions:**

The deficiency of vitamin B12 and folate is still prevalent among the Chinese women in northwest China. Vitamin B12 deficiency could be more serious and the improvement of poor vitamin B12 status should be invoked when practicing the supplementation of folate against the neural tube defects in northwest China.

## Introduction

There was a widespread low folate status around China, especially in the region of northern China [1]–[4]. A previous study showed that the mean plasma folate in the adults of northern China was about 8.4 nmol/L, which was significantly lower than 16.7 nmol/L in southern region controlling for age, gender, season and so on [3]. However, most of studies on folate status focused on specific regions and women of childbearing age so it was difficult to estimate the number of folate deficient people in China. Based on limited data, the prevalence of folate deficiency among Chinese women of childbearing age varied from 1% to 20% [1]. Currently available evidences indicated that folate deficiency was associated with increasing risk of neural tube defects (NTD) [5], [6]. Folic acid supplementation during the periconceptional period was regarded as an effective strategy against NTD although the mandatory folic acid fortification to reduce NTD rates was introduced in some countries [5]–[7]. In China the policy of supplementation of folic acid played an important role in preventing NTD [8]. However, magnitude of reduction in NTD rate much varied with regions of China. In Shaanxi province, a high NTD incidence area of China, NTD rate had been reduced to about 1.3‰ in 2007 but since then the rate persisted [9]. The reason might be complicated. Other micronutrient deficiency such as vitamin B12 could be taken into account in addition to lower compliance of the folate supplementation. Vitamin B12 plays an essential role in folate metabolism and the enzyme methionine synthase catalyzes the vitamin B12-dependent conversion of homocysteine and 5-methyltetrahydrofolate to methionine and tetrahydrofolate [10]. Therefore, vitamin B12 deficiency could impair the folate-mediated one-carbon metabolic pathway to present as folate deficiency or to exacerbate folate deficiency. There was increasing evidence that poor maternal vitamin B12 status may increase the risk of adverse pregnancy outcomes including NTD [11]–[14]. A Canadian study found a 3-fold increase in the risk of NTDs in mothers who had vitamin B-12 status in the lower quartile, regardless of folate fortification [13]. It implied that deficiency of vitamin B12 might be an independent factor resulting in NTD. Moreover, folate supplementation could mask vitamin B12 deficiency [10]. Suboptimal vitamin B12 status is common in many parts of the world [11], [15]. Scientific evidence on vitamin B12 status of Chinese population was limited. Compared with Chinese adults in southern China, adults from the northern regions presented low vitamin B12 status and prevalence of deficiency was about 21% [16]. Accordingly we measured plasma folate and vitamin B12 status of a representative sample of Chinese women living in Shaanxi province. We tried to profile status of vitamin B12 and folate among Chinese women and investigate the relationship between the two vitamins. These data would be helpful in determination of micronutrient supplementation programs in China.

## Methods

### Study setting

Shaanxi province is located in northwest China geographically with such three regions as northern, middle and southern Shaanxi. The natural environment, culture and lifestyle vary significantly across the three regions. Northern Shaanxi is in the loess plateau of China with semi-arid temperate climate. Middle Shaanxi is situated in the alluvial plains and north temperate monsoon climate predominates. Southern Shaanxi is located in the south of Qinling Mountain which is the main boundary between the south and north of China. It is humid and sub-tropical typically. The resident population is 37.5 million people in Shaanxi and about 30% of them live in urban areas. Compared with the eastern China, Shaanxi is a developing province in economy and society. In 2012 the provincial GDP amounted to 23.6 billion US dollars, ranking sixteenth in China [17].

### Sample and subjects

A population-based cross-sectional study was conducted between August and September, 2008. The subject was the Chinese women aged 10–49 years living in Shaanxi. Women were excluded if they were breastfeeding or had breast-fed within the last 12 months, or had a serious or chronic illness. A stratified multistage random sampling method was adopted to obtain the sample. Women were recruited from the urban and rural areas, respectively. In rural areas, 20 counties were selected randomly from 80 counties of Shaanxi according to population size. For each sampled county, one township was selected randomly and one village selected was randomly from the sampled township. Finally the rural women were selected randomly in sampled village. In urban areas, 10 districts were selected randomly from 24 urban districts. One community was obtained randomly from each sampled district and the urban women were selected in each sampled community. A sample size of 1100 was estimated assuming a prevalence of folate deficiency of 15%, a relative error of 15%, α = 0.05, accounting for an expected 20% nonresponse rate. The proportion of urban to rural subjects in the sample was determined as about 30%, which was close to a proportion of urban to rural population in Shaanxi province.

### Data collection

The subjects were interviewed face to face by the trained staff with a standard questionnaire to collect the information including social-demographical information of women, household background and the woman's health and reproductive history. Anthropometric data of the women was obtained using standard measuring apparatus. Five milliliters of venous blood was taken by venepuncture on arm into tubes containing EDTA and the plasma was isolated on site. All specimens were transported on dry ice to the laboratory center of college of public health, Xi'an Jiaotong University and kept in the refrigerators with −70°C. Measurement of plasma vitamin B12 and folate were performed with immunoassay method using the kit from MP Biomedicals LLC Ltd within two months after collection of blood specimens. The plasma folate concentration of <3 ng/mL was used to indicate folate deficiency and 3–6 ng/mL for marginal folate status. Vitamin B12 status was defined as deficient based on plasma vitamin B12 concentration of <200 pg/mL and 200–299 pg/mL for marginal vitamin B12 status [18].

### Survey quality control

Three field-working teams were established according to geographic distribution of sampled sites. Each team consisted of 4 members who accounted for blood collecting, plasma isolation, subject interview, and anthropometry. Staff from county health bureau or maternal and child health care station coordinated activities of each team in each sampled site. And doctors from village clinics or township hospitals were involved in the field-working. Before the formal survey, a training meeting was launched in Xi'an and all members of the teams were trained. The University Health Science Center made training materials and was in charge of training including discussion and practice. After each interview, investigators checked all information in questionnaire. Key member in the team accounted for checking all questionnaires of each investigating site. If errors happened, repeated interview was carried out. All measuring apparatus were corrected before measurement and standard procedures were followed.

### Data analysis

A database was established by duplicate entries using EpiData 3.02 software (The EpiData Association, Odense, Denmark). Prior to data analysis, data cleaning and quality check were performed. Skewed distribution of vitamin B12 and folate was observed, so median and interquartile range was used as descriptive statistics. Differences between groups for the selected variables were determined using *x* ^2^ test for categorical variables and Kruskal-Wallis test for continuous variables. Kernel density method was employed to describe the distribution of vitamin B12 and folate. The distribution curves by age were plotted with epanechnikov kernel function and width of 0.5. Quantile regression model was used to investigate the relationship between vitamin B12 and folate controlling for potential affecting factors including age, BMI, education level, geographical region, source of family income and morbidity of cold. The model in this setting would allow the impact of the folate status (deficiency or not) to vary along the whole range of vitamin B12 value. Seventy-three different conditional quantile functions were estimated by the variable of residence (urban vs. rural), starting with the 0.05 quantile and proceeding in 0.0125 increments until the 0.95 quantile (i.e., 0.05, 0.0625, 0.75, 0.875, 0.1 … 0.95). A Markov chain marginal bootstrap was used to compute 95% confidence intervals (CIs) of coefficient for every quantile estimate. Coefficients with 95% CIs of folate status were plotted across the different quantile of vitamin B12. Negative coefficient at each quantile indicated decreasing amount of vitamin B12 of women with folate deficiency compared with those with normal status. The estimated coefficients for selected quantile with P-value were reported with the coefficient by a typical regression using ordinary least squares (OLS) to provide a basis for comparison with the quantile regression. A P-value <0.05 was considered statistically significant. All statistical analyses were performed using the STATA statistical software package version 9 (StataCorp, College Station, TX, USA).

### Ethics Statement

The study complied with the Declaration of Helsinki and was approved by the Ethical Committee of Science of Medical center, Xi'an Jiaotong University. The written informed consent had been obtained from the study participants, and the mothers of the girls aged less than 18 years provided the written consent on behalf of these girls.

## Results

### The characteristics of the participants

Totally 1193 women were investigated. Among them 23 women were excluded for incomplete or missing value in the vitamins and 1170 data were used for analysis finally. Demographic characteristics were shown in Table 1. All women were ethnically Han Chinese. Rural participants accounted for 67.9%. Geographically, 63.3% of the participants lived in central Shaanxi. The proportion of participants for each age group was about 20% apart from age group of 20 years. The mean of BMI was 18.4 for the women aged 10–14.99 years, 19.8 for the women aged 15–19.99 years and 22.7 for the women aged over 20 years. The women aged over 20 years had 9.51 years of schooling. Almost one-fourth of participants' family lived on farming and 23.5% on migrant working. In remaining families, working in government institutions or company was main source of income. About health status, 16.8% of the women had caught a cold within 2 weeks before investigating. The prevalence of anemia was about 27.1% (Table 1).

**Table 1:** Characteristics of the female participants in the study.

Variables	n	Outcome
Residence (%)		
Rural	794	67.9
Urban	376	32.1
Geography (%)		
North	233	19.9
Central	741	63.3
South	196	16.8
Age group (years) (%)		
10−	276	23.6
15−	280	23.9
20−	117	10.0
30−	267	22.8
40−	230	19.7
BMI by age 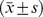 *		
10−	276	18.4±2.75
15−	264	19.8±2.24
20+	614	22.7±3.11
Years of schooling by age 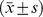 *		
10−	274	7.44±1.42
15−	261	9.21±1.45
20+	609	9.51±4.16
Source of family income (%)*		
Farm only	285	24.8
Migrant working	270	23.5
Government institutions	139	12.1
State-owned Company	214	18.7
Private company	239	20.8
History of catching a cold within 2 weeks (%)*		
Yes	197	16.8
No	956	81.7
Anemia (%)*		
Yes	317	27.1
No	847	72.4

^*^Few data were missing.

### The average status and distribution of vitamin B12 and folate

**Figure 1:**
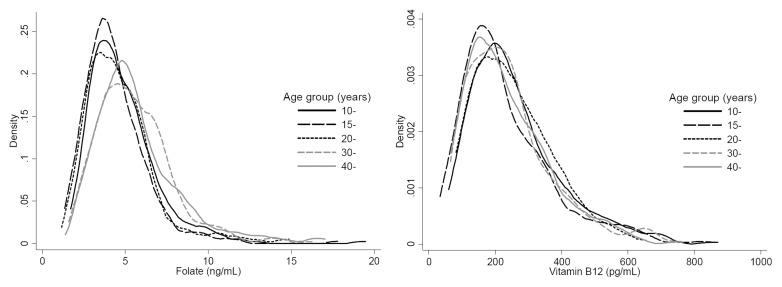
Distribution of vitamin B12 and folate by age among Chinese women in Shaanxi province.

Table 2 showed that the median value was 214.5 pg/mL for vitamin B12 and 4.6 ng/mL for folate among Chinese women in Shaanxi province. The urban women had a significantly higher vitamin B12 (254.1 vs. 195.9 pg/mL) but lower folate (4.4 vs. 4.7 ng/mL) than rural women. The status of two vitamins varied geographically (P<0.001). The women in south and central Shaanxi presented no difference in vitamin B12, but had higher vitamin B12 level than the women in north Shaanxi. The women living in north and middle Shaanxi had a similar level of folate, which were lower significantly compared with the women in south Shaanxi. Figure 1 indicated that the distribution of folate moved upward with age increase and the women aged over 30 years presented higher folate significantly (P<0.001). However, there might be a complicated relationship between vitamin B12 and age. The women aged over 30 years had significantly lower vitamin B12 (P<0.001). Obviously the adolescent girls had a lower status in both folate and vitamin B12 (P<0.001).

**Table 2:** The median and distribution of vitamin B12 and folate by the residence, geographical environment and age.

		Vitamin B12 (pg/mL)					Folate (ng/mL)			
		Median (P_25_, P_75_)	n(%)				Median (P_25_, P_75_)	n(%)		
	n		<100	100-	200-	300-		<3	3-	6-
All women	1170	214.5	125	407	315	323	4.6	172	694	304
		(148.1,311.4)	(10.7)	(34.8)	(26.9)	(27.6)	(3.5,6.0)	(14.7)	(59.3)	(26.0)
Residence*										
Rural	794	195.9	105	308	208	173	4.7	109	468	217
		(137.4,283.2)	(13.2)	(38.8)	(26.2)	(21.8)	(3.6,6.1)	(13.7)	(58.9)	(27.3)
Urban	376	254.1	20	99	107	150	4.4	63	226	87
		(182.2,359.9)	(5.3)	(26.3)	(28.5)	(39.9)	(3.4,5.9)	(16.8)	(60.1)	(23.1)
Geography†										
North	233	188.7	40	87	64	42	4.5	34	147	52
		(126.5,274.1)	(17.2)	(37.3)	(27.5)	(18.0)	(3.5,5.7)	(14.6)	(63.1)	(22.3)
Central	741	219.6	73	255	190	223	4.4	126	450	165
		(152.2,324.1)	(9.9)	(34.4)	(25.6)	(30.1)	(3.3,5.8)	(17.0)	(60.7)	(22.3)
South	196	226.8	12	65	61	58	5.8	12	97	87
		(164.2,323.0)	(6.1)	(33.2)	(31.1)	(29.6)	(4.2,7.2)	(6.1)	(49.5)	(44.4)
Age group (years)‡										
10-	276	233.7	23	85	84	84	4.4	39	175	62
		(163.3,329.6)	(8.3)	(30.8)	(30.4)	(30.4)	(3.4,5.7)	(14.1)	(63.4)	(22.5)
15-	280	197.7	37	110	59	74	4.0	57	183	40
		(140.9,304.4)	(13.2)	(39.3)	(21.1)	(26.4)	(3.1,5.3)	(20.4)	(65.3)	(14.3)
20-	117	229.8	8	41	33	35	4.3	22	71	24
		(154.4,333.9)	(6.8)	(35.0)	(28.3)	(29.9)	(3.2,5.5)	(18.8)	(60.7)	(20.5)
30-	267	216.8	29	88	83	67	5.0	30	134	103
		(144.3,302.4)	(10.9)	(33.0)	(31.1)	(25.1)	(3.9,6.7)	(11.2)	(50.2)	(38.6)
40-	230	204.9	28	83	56	63	5.1	24	131	75
		(144.2,309.2)	(12.2)	(36.1)	(24.3)	(27.4)	(4.0,6.6)	(10.4)	(57.0)	(32.6)

^*^Compared with rural women, urban women had higher vitamin B12 (P<0.001) and lower folate (P = 0.029).

† The vitamin B12 in south and central Shaanxi was not different statistically (P = 0.379) but higher than that of north Shaanxi (P<0.001). The folate in north and central Shaanxi was not different statistically (P = 0.576) but lower than that of south Shaanxi (P<0.001).

‡ There were significantly lower vitamin B12 and folate among the women aged 15-19.99 years (P<0.001).

### Prevalence of deficiency of vitamin B12 and folate

**Figure 2:**
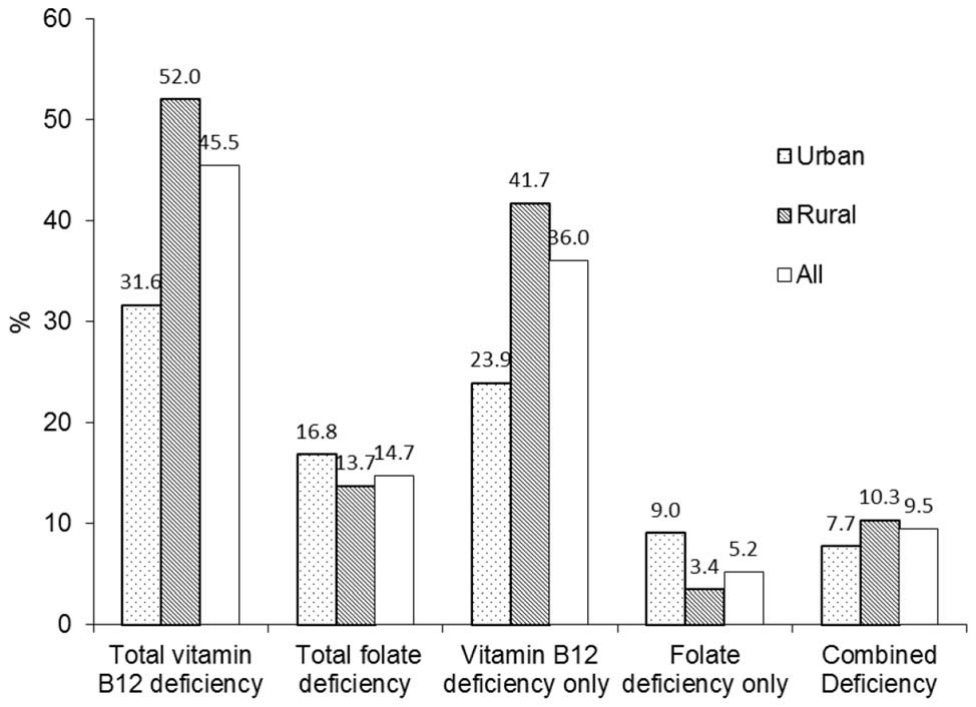
Prevalence of deficiency in vitamin B12 and folate among Chinese women in Shaanxi province.

Figure 2 showed the prevalence of deficiency of two vitamins. Total prevalence of deficiency was 45.5% (95% CI: 42.6%∼48.4%) for vitamin B12 and 14.7% (95% CI: 12.6%∼16.8%) for folate. About 36% of the women presented vitamin B12 deficiency alone and 5.2% belonged to folate deficiency alone. Prevalence of combined deficiency of both vitamins was about 9.5%. The total prevalence of vitamin B12 deficiency was higher in rural women than urban women (52.0% vs. 31.6%, P = 0.004) but there was no difference in folate deficiency between them (13.7% vs. 16.8%, P = 0.172). Compared with urban women, rural women showed a higher prevalence of vitamin B12 deficiency alone (41.7% vs. 23.9%, P<0.001) but lower prevalence of folate deficiency alone (3.4% vs 9.0%, P = 0.001). There was no difference in prevalence of combined two vitamins deficiency found between rural and urban women (10.3% vs. 7.7%, P = 0.156).

Based on the distribution of vitamin B12 in Table 2, rural women showed significantly lower vitamin B12 than urban women (Table 2, P<0.001). It was not different in distribution of folate between rural and urban women (P = 0.185). The women living in south Shaanxi were in higher status in both vitamins, compared with the women in north and central Shaanxi (P<0.001). It was worth noting that more than 25% of the women were in the marginal vitamin B12 status and almost 60% of the women in the marginal status of folate.

### Relationship between vitamin B12 and folate

**Figure 3:**
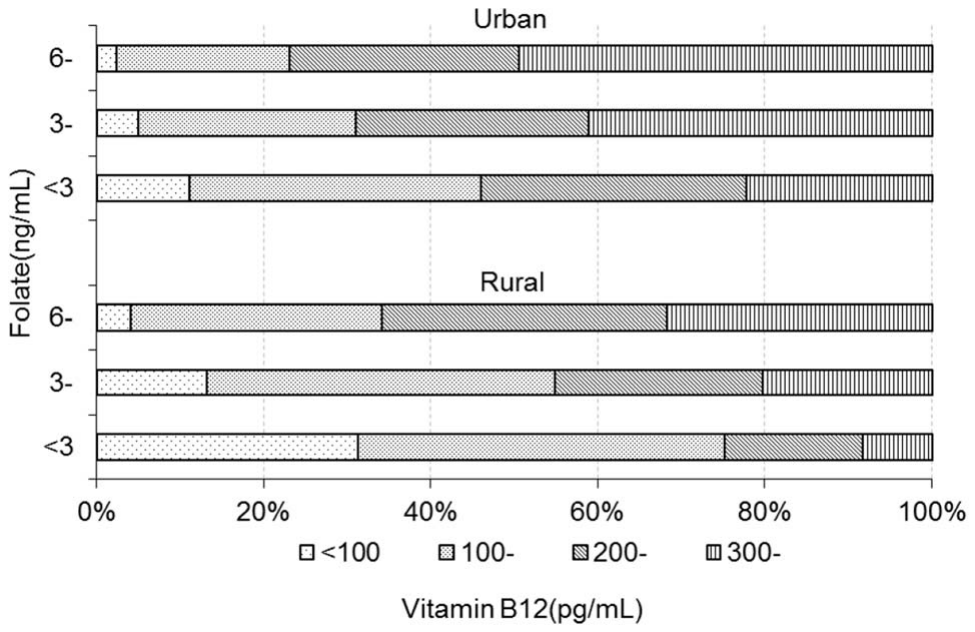
The relationship between the vitamin B12 and folate among Chinese women in Shaanxi province.

**Figure 4:**
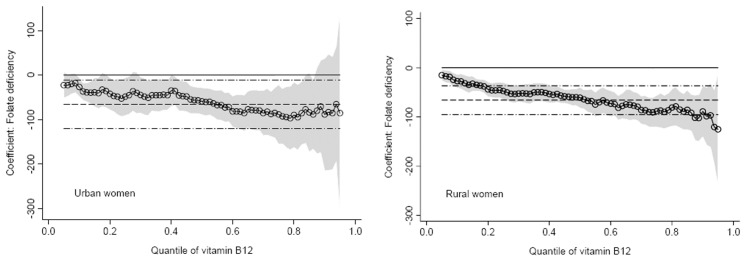
Quantile regression and ordinary least squares (OLS) estimates by residence (urban vs rural): folate deficiency and vitamin B12. Circles represent quantile regression estimates, and the shaded areas around the quantile regression estimates were the 95% confidence intervals. The dashed line was OLS estimate, and the dash-dotted lines showed the OLS 95% confidence interval. Estimates shown here compared folate deficiency with no-deficiency.

Figure 3 showed that the women with folate deficiency might be a high risk of being deficient in vitamin B12. About 75.2% of the rural women with folate deficiency were vitamin B12 deficiency. Among urban women with the folate deficiency, about 46% was deficient in vitamin B12 and 31.7% belonged to the marginal vitamin B12 status. Quantile regression model was used to investigate quantitatively the relationship between vitamin B12 and folate status controlling for the potential affecting factors. Figure 4 indicated the change of coefficient of folate status across 73 different quantiles of vitamin B12. In rural areas, the coefficients were negative and decreased with quantiles and even at higher quantiles the women with folate deficiency had lower vitamin B12 significantly compared with those with no deficiency. In urban areas, the similar trend for the coefficients was observed but at higher quantiles (>85% quantile) there was no significant difference found in vitamin B12 between women with folate deficiency and those without deficiency. Table 3 presented the results for a selected set of quantiles: 0.05, 0.10, 0.25 (lower vitamin B12 level), 0.45, 0.50 (medium vitamin B12 level), 0.75, 0.85, 0.95 (higher vitamin B12 level). Table 3 also showed the multiple linear regression (OLS) estimates at last column. In the rural women with lower vitamin B12 level, vitamin B12 decreased by 15–47 pg/mL in the women with folate deficiency compared with those without deficiency. Even in the rural women with higher vitamin B12 level, the women with folate deficiency had a significant decrease of 88–125 pg/mL in vitamin B12 compared with those without deficiency. The similar situation was found in the urban women but there was no significant difference in vitamin B12 between the women with and without folate deficiency when they were at higher vitamin B 12 level. OLS model estimated that mean difference in vitamin B12 was about 65 pg/mL between the women with and without folate deficiency regardless of urban or rural women.

**Table 3:** Estimated coefficients from and multiple liner regression quantile regression for selected quantiles: vitamin B12 and folate status by residence.*

		Quantile								
Folate deficiency		0.05	0.10	0.25	0.45	0.50	0.75	0.85	0.95	OLS
Rural										
	Coefficient†	−15.00	−27.19	−47.36	−57.98	−60.69	−88.24	−85.34	−125.08	−65.74
	*P*-value	0.011	<0.001	<0.001	<0.001	<0.001	<0.001	<0.001	0.037	<0.001
Urban										
	Coefficient†	−22.40	−26.97	−48.40	−48.17	−58.87	−88.65	−83.95	−85.53	−65.61
	*P*-value	0.136	0.013	0.026	0.006	<0.001	0.01	0.065	0.458	0.018

^*^Quantile regression model and ordinary least squares (OLS) were used for estimates. In the models the dependent variable was vitamin B12 concentration and folate status (deficiency or not) were regarded as the independent variable controlling for potential affecting factors including age, BMI, education level, geographical region, source of family income and morbidity of cold.

† The coefficient here meant the change of vitamin B12 of women with folate deficiency compared with those with normal status of folate.

## Discussion

This study was conducted in Shaanxi province of China with a stratified multistage random sampling method based on population distribution, so the sample was a representative of the Shaanxi women and the results from the study also reflected the status of folate and vitamin B12 of Chinese women to some extent. The study outlined the epidemiological features about folate and vitamin B12 among the women in Shaanxi, which implied that the deficiency of folate and vitamin B12 was still prevalent among the Chinese women.

Levels of folate and vitamin B12 were low among the women in Shaanxi, and about 14.7% of the women were deficient in folate and 45.5% of the women were deficient in vitamin B12. Significant regional differences were found. Firstly, the urban women had a significantly higher vitamin B12 (254.1 vs. 195.9 pg/mL) but lower folate (4.4 vs. 4.7 ng/mL) than rural women. Such urban-rural difference was also observed among other Chinese woman population [3]. Secondly, geographical difference in folate and vitamin B12 was also obvious, which showed that the women living in southern Shaanxi presented higher folate and vitamin B12 level significantly than those in northern areas. Southern Shaanxi is located in the south of Qinling Mountain which is a boundary between the south and north of China, and the climate and lifestyle in southern Shaanxi is close to typical areas of southern China such as Sichuan [17]. Therefore, this result supported partly the fact of significant difference in the folate and vitamin B12 between the northern and southern China [3]. The geographical difference in folate and vitamin B12 status was consistent with the local lifestyle of Shaanxi and there were the greater availability of fresh vegetables and animal-source foods in the south than the north [17]. Compared with other areas of China, the prevalence of deficiency in folate among Shaanxi women was not very higher, which might be in the middle level. However, notably more than half of women (59.3%) were exposed to lower status of folate (3–6 ng/mL) and the plasma folate in the Shaanxi women was only one third of the American women [19], which suggested that supplementation of folate to the Shaanxi women should be recommended strongly. Around the world, the prevalence of vitamin B12 deficiency varied much from 14.8% to 54.5% [20]–[23]. Clearly Shaanxi women had a very higher prevalence of deficiency in vitamin B12; especially it was worse in the rural women. Now no national data could be available about vitamin B12 status in China and the magnitude of vitamin B12 deficiency had not been clear. Based on the current local data [4], the prevalence of vitamin B12 deficiency in the Chinese adults was about 4% in the south and 21% in the north. Our study implied that Shaanxi women might be in worse status of vitamin B12.

We also found that the status of folate and vitamin B12 was significantly related to the age. The results showed that the old women had a higher status of folic acid than younger women. The women aged 15–19.99 years had a lowest folate (the median: 4.0 ng/mL) and the women aged 20–29.99 years followed (the median: 4.3 ng/mL). These two groups of women were in child-bearing period when enough folate was required for preparation of pregnancy. So low folate level made them being high risk of NDTs. As for vitamin B12, the age distribution was different. Both younger and older women were in lower status of vitamin B12. Vitamin B12 absorption tended to decrease with age, which might explain some of the difference between age groups [24]. However, we found that younger women, especially adolescent girls, were at the higher risk of being lower status of both folate and vitamin B12. Adolescent girls were in the stage of growth and lower level of vitamins might affect their health adversely. And it suggested that the supplementation of folate and vitamin B12 should focus on the younger female population.

In Shaanxi, only 5.2% of women presented folate deficiency alone but 36% of women were deficient alone in vitamin B12, which suggested that the vitamin B12 deficiency might be predominant and serious, especially in the rural women. Nearly 10% of the women were deficient in both folate and vitamin B12. And among the women with folate deficiency, more than half of the women were deficient in vitamin B12. Further, we investigated quantitative difference in vitamin B12 between the women with and without folate deficiency by using quantile regression model. Compared with the women without folate deficiency, the women with folate deficiency were down 15–125 pg/mL in vitamin B12 level, and averagely about 65 pg/mL. It meant that the women with folate deficiency might be in higher risk of being low vitamin B12. The periconceptional supplementation of folic acid had been practiced in the many countries including China and consequently NTDs were prevented or controlled significantly. Since mid-1990s China has encouraged supplementation of folic acid during the periconceptional period against NTDs and began to provide all women who plan to have a child of free supplements in 2009 [8]. The overall prevalence of NTDs had been decreased from 1.2‰ in 2000 to 0.45‰ in 2011 [25]. But up to now Shaanxi province had still a prevalence of NTDs of 1.3‰ [9] and low vitamin B12 could account for it to some extent. Vitamin B12 played an essential role in folate metabolism and vitamin B12 deficiency could impair the folate-mediated one-carbon metabolic pathway to present as folate deficiency or to exacerbate folate deficiency [10]. The study by Molloy AM et al. found that the mothers of children affected by NTDs had significantly lower B12 status and blood vitamin B12 concentrations of <250 pg/mL during pregnancy was associated with the highest risks [14]. Literatures reported that the hyperhomocysteinemia, which was caused by deficiency in folate and vitamin B12, might be associated with neural tube defects, cardiovascular disease, and mental disorders [6], [19], [26]–[29]. A study in China by Ling Hao et al. found that vitamin B12 deficiency alone was associated with 4-fold increased risk of being hyperhomocysteinemia and the individuals deficient in both folate and vitamin B-12, had 38-fold higher risk of the hyperhomocysteinemia [16]. Therefore, higher prevalence of vitamin B12 deficiency was associated possibly with NTDs in Shaanxi and further assessment should be required. Moreover, vitamin B12 deficiency often presented as folate deficiency and elevated intakes of folate could interfere with a clinical diagnosis of vitamin B12 deficiency, which implied that folate supplementation could mask vitamin B12 deficiency [10]. This problem also should be paid more attention in the practice of supplementation of folic acid in Shaanxi women.

One of the important causes about lower status of folate and vitamin B12 could be poor dietary pattern [23], [30]. Based on our dietary survey among rural women in Shaanxi, the intake of animal protein intake was very low among Shaanxi women [31]. And the intake of fresh vegetables (296.2 g/day) and fruits (25g/day), and animal-source foods (19.4 g/day) was lower significantly than Chinese RNI/AI [32]. Simple diet rich in carbohydrate was a predominant pattern among Shaanxi women [33]. So enriching the dietary pattern and increasing intake of animal foods might be an important way to improve the status of vitamin B12 and folate of Chinese women.

In this study, one limitation had to be addressed. Our survey was about the health status of Shaanxi women and only such blood indicators as folate, vitamin B12 and hemoglobin were measured. Unfortunately, the metabolic markers of folate and vitamin B12, homocysteine (tHcy), methylmalonic acid (MMA) and holotranscobalamin (holoTC), had not been measured. Studies indicated that these markers increased or decreased when the status of plasma folate and vitamin B12 were low [16], [34], [35]. The elevated tHcy and MMA were found to be related to cognitive decline but high holoTC level was protective against cognitive decline [36], [37]. However, tHcy and MMA started to increase at vitamin B12 levels above the typical cut-off value (200 pg/mL), and they increased quickly when vitamin B12 decreased from 400 to 200 pmol/L [38], which suggested that the change of the markers related to the impairment of functioning of the nervous system had already begun before the occurrence of deficiency in vitamin B12 or folate. Therefore, in the view of public health, the detection of such metabolic markers might have an important significance to the control and prevention of neurological disease although more epidemiological evidence still were required [36], [39]. Our study found that about 60% of the women were in marginal folate status and 27% in marginal vitamin B12 status and so these women should be paid more attention. Further assessment of the metabolic markers of folate and vitamin B12 could be essential among such female population. Another limitation was that the findings from this study could not be extrapolated to the rest of China, although the sample was representative of Shaanxi province. China is a country that is geographically, climatically and ethnically diverse. Clearly these differences could affect food supply, dietary practices, and consequently folate and vitamin B12 intakes. Geographical difference in folate and vitamin B12 was significant between south and north of China, which might result from different dietary patterns and climate. Moreover, we did not test red blood cell folate in this survey, which could influence accurate estimation of folate level. Nevertheless, our findings provided strong evidence that the deficiency in folate and vitamin B12 was prevalent among Shaanxi women, and especially vitamin B12 deficiency was more serious. Younger women were a high-risk population of being deficient in folate and vitamin B12. We suggested that the control of vitamin B12 deficiency should be considered when practicing the supplementation of folate against NTD in China.
